# Driving Under the Influence of Marijuana and Illicit Drugs Among Persons Aged ≥16 Years — United States, 2018

**DOI:** 10.15585/mmwr.mm6850a1

**Published:** 2019-12-20

**Authors:** Alejandro Azofeifa, Bárbara D. Rexach-Guzmán, Abby N. Hagemeyer, Rose A. Rudd, Erin K. Sauber-Schatz

**Affiliations:** ^1^Consultant, Washington, DC; ^2^Consultant, San Juan, Puerto Rico; ^3^Applied Epidemiology Fellowship, Council of State and Territorial Epidemiologists, Atlanta, Georgia; ^4^Division of Injury Prevention, National Center for Injury Prevention and Control, CDC.

In the United States, driving while impaired is illegal. Nonetheless, an estimated 10,511 alcohol-impaired driving deaths occurred in 2018.* The contribution of marijuana and other illicit drugs to these and other impaired driving deaths remains unknown. Data from the Substance Abuse and Mental Health Services Administration’s National Survey on Drug Use and Health (NSDUH) indicated that in the United States during 2014, 12.4% of all persons aged 16–25 years reported driving under the influence of alcohol, and 3.2% reported driving under the influence of marijuana (*1*). The impairing effects of alcohol are well established, but less is known about the effects of illicit substances or other psychoactive drugs (e.g., marijuana, cocaine, methamphetamines, and opioids, including heroin). This report provides the most recent national estimates of self-reported driving under the influence of marijuana and illicit drugs among persons aged ≥16 years, using 2018 public-use data from NSDUH. Prevalences of driving under the influence of marijuana and illicit drugs other than marijuana were assessed for persons aged ≥16 years by age group, sex, and race/ethnicity. During 2018, 12 million (4.7%) U.S. residents reported driving under the influence of marijuana in the past 12 months; 2.3 million (0.9%) reported driving under the influence of illicit drugs other than marijuana. Driving under the influence was more prevalent among males and among persons aged 16–34 years. Effective measures that deter driving under the influence of drugs are limited (*2*). Development, evaluation, and further implementation of strategies to prevent alcohol-impaired,^†^ drug-impaired, and polysubstance-impaired driving, coupled with standardized testing of impaired drivers and drivers involved in fatal crashes, could advance understanding of drug- and polysubstance-impaired driving and support prevention efforts.

NSDUH annually collects information about the use of illicit drugs, alcohol, and tobacco among the noninstitutionalized U.S. civilian population aged ≥12 years via household face-to-face interviews using a computer-assisted personal interviewing system.^§^ Respondents aged <16 years were excluded from this analysis because they are typically too young to drive. Unweighted sample sizes for the 2018 survey cycle included 47,570 respondents aged ≥16 years. Driving under the influence of marijuana was defined as an affirmative response to the question “During the past 12 months, have you driven a vehicle while you were under the influence of marijuana?” Driving under the influence of illicit drugs other than marijuana was defined as an affirmative response to one or more of the questions (each asked separately) that asked about each illicit drug: “During the past 12 months, have you driven a vehicle while you were under the influence of (cocaine, hallucinogens, heroin, inhalants, methamphetamine)”? Public-use NSDUH data on driving under the influence of marijuana and illicit drugs other than marijuana were examined by sex, age group, and race/ethnicity. Data were weighted to provide nationally representative estimates. Statistical analyses were performed using SAS (version 9.4; SAS Institute). Prevalence measures and 95% confidence intervals (CIs) were determined for each response category.

During 2018, the overall prevalence of driving under the influence of marijuana (4.7%) exceeded that of driving under the influence of illicit drugs other than marijuana (0.9%) among persons aged ≥16 years (Table). This pattern persisted when the data were stratified by sex, race/ethnicity, and age group. The prevalences of driving under the influence of marijuana and driving under the influence of illicit drugs other than marijuana were higher among males (6.2%, 1.3%, respectively) than among females (3.2%, 0.5%, respectively). The prevalence of driving under the influence of marijuana was highest among non-Hispanic multiracial persons (9.2%). The prevalence of driving under the influence of marijuana ranged from 0.6% among persons aged ≥65 years to 12.4% among persons aged 21–25 years; the second highest prevalence (9.2%) was reported among persons aged 16–20 years (Figure). The highest reported prevalences of driving under the influence of illegal drugs other than marijuana were among persons aged 21–25 years (1.9%) and 26–34 years (1.9%).

**TABLE:** Number and percentage of all persons aged ≥16 years* who reported driving a vehicle while under the influence of marijuana or illicit drugs other than marijuana^†^ in the past year, by demographic characteristics — National Survey on Drug Use and Health, United States, 2018

Characteristic	Marijuana		Illicit drugs other than marijuana	
	No. who reported driving under the influence (x 1,000)	% (95% CI)	No. who reported driving under the influence (x 1,000)	% (95% CI)
**Sex**				
Male	7,711	6.2 (5.9–6.6)	1,578	1.3 (1.1–1.5)
Female	4,249	3.2 (2.9–3.5)	722	0.5 (0.4–0.7)
**Race/Ethnicity^§^**				
White	7,913	4.9 (4.5–5.2)	1,601	1.0 (0.9–1.1)
Black	1,576	5.1 (4.5–5.7)	182	0.6 (0.3–0.9)
American Indian/Alaska Native	72	4.9 (2.7–7.1)	18	1.2 (0.2–2.2)
Hawaiian/Other Pacific Islander	35	3.6 (0.9–6.3)	13	1.4 (0.0–3.3)
Asian	336	2.3 (1.2–3.4)	74	0.5 (0.2–0.9)
Multiracial	427	9.2 (6.3–12.1)	50	1.1 (0.5–1.6)
Hispanic	1,602	3.8 (3.2–4.4)	362	0.9 (0.6–1.1)
**Total**	**11,960**	**4.7 (4.4–4.9)**	**2,300**	**0.9 (0.8–1.0)**

**Abbreviation:** CI = confidence interval.

* Numbers and percentages are weighted to represent the 2018 U.S. civilian, noninstitutionalized population and are not mutually exclusive.

^†^ Illicit drugs other than marijuana in this analysis are cocaine, hallucinogens, heroin, inhalants, and methamphetamines.

^§^ Whites, blacks, American Indian/Alaska Natives, Hawaiian/Other Pacific Islanders, Asians, and multiracial persons were non-Hispanic; Hispanic persons could be of any race.

**FIGURE:**
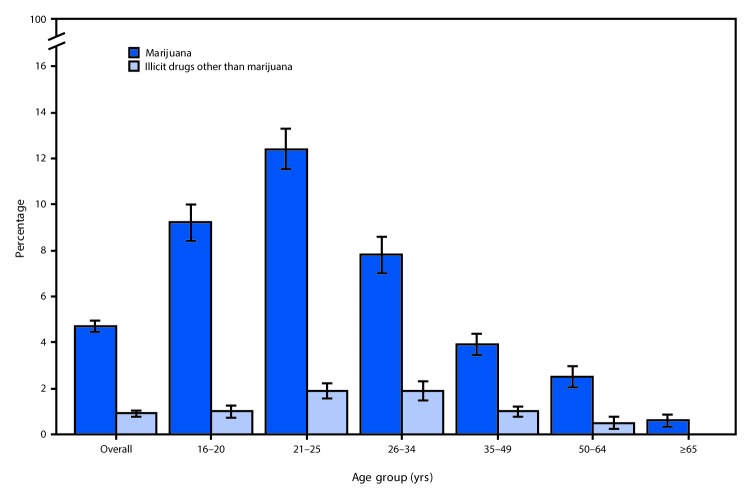
Percentage of all persons aged ≥16 years* who reported driving a vehicle under the influence of marijuana or illicit drugs other than marijuana^†^^,^^§^^,^^¶^ in the past year, by age group** — National Survey on Drug Use and Health, United States, 2018 * Percentages are weighted to represent the 2018 U.S. civilian, noninstitutionalized population. ^†^ Illicit drugs other than marijuana in this analysis include cocaine, hallucinogens, heroin, inhalants, and methamphetamines. ^§^ Not mutually exclusive. ^¶^ Estimated percentage of adults aged ≥65 years who reported driving under the influence of illicit drugs other than marijuana was <0.02% and thus not shown. ** With 95% confidence intervals indicated by error bars.

## Discussion

Although 4.7% of the U.S. population aged ≥16 years reported driving under the influence of marijuana and 0.9% reported driving under the influence of illicit drugs other than marijuana, these estimates are lower than the 8.0% (20.5 million) who reported driving under the influence of alcohol in 2018 (NSDUH, unpublished data, 2019). The highest prevalence of driving under the influence of marijuana was among persons aged 21–25 years. The second highest was among the youngest drivers (those aged 16–20 years), who already have a heightened crash risk because of inexperience^¶^; thus, their substance use is of special concern. In a study of injured drivers aged 16–20 years evaluated at level 1 trauma centers in Arizona during 2008–2014 (*3*), 10% of tested drivers were simultaneously positive for both alcohol and tetrahydrocannabinol, the main psychoactive component of marijuana. Data from the 2018 NSDUH indicate a high prevalence (34.8%) of past-year marijuana use among young adults aged 18–25 years (*4*). Studies have reported that marijuana use among teenagers and young adults might alter perception, judgement, short-term memory, and cognitive abilities (*5*). Given these findings, states could consider developing, implementing, and evaluating targeted strategies to reduce marijuana use and potential subsequent impaired driving, especially among teenagers and young adults.

Research has determined that co-use of marijuana or illicit drugs with alcohol increases the risk for driving impairment (*5*,*6*). The use of these substances has been associated with impairment of psychomotor and cognitive functions while driving (*6*,*7*). In addition, previous research has demonstrated evidence of a statistical association between marijuana use and increased risk for motor vehicle crashes; however, methodologic limitations of studies limit inference of causation (*8*). Scientific studies have been unable to link blood tetrahydrocannabinol levels to driving impairment (*8*), and the effects of marijuana in drivers likely varies by dose, potency of the product consumed, means of consumption (e.g., smoking, eating, or vaping), length of use, and co-use of other substances, including alcohol. Additional data are needed to clarify the contribution of drug and polysubstance use to impaired driving prevalence and the resulting crashes, injuries, and deaths.

A national roadside survey using biochemical specimens among drivers aged ≥16 years found that during 2013–2014, the percentages of weekend nighttime drivers who tested positive for alcohol, marijuana (i.e., tetrahydrocannabinol) and illicit drugs were 8.3%, 12.6%, and 15.1%, respectively (*9*), although a positive test does not necessarily imply impairment. Collecting and testing biologic specimens (e.g., blood or oral fluids) currently required to test for drugs has challenges, including, in some circumstances, the need for a judge to order collection and testing (which can delay roadside testing, thus allowing drug levels to drop with time); variation in substances tested and methodology used by different toxicology laboratories; and the current state of development of oral fluid testing. The increased use of marijuana and some illicit drugs in the United States (*4*) along with the results of this report, point to the need for rapid and sensitive assessment tools to ascertain the presence of and impairment by marijuana and other illicit drugs. In addition, adoption and application of standards for toxicology testing and support for laboratories to implement recommendations are needed to improve understanding of the prevalence of drug- and polysubstance-impaired driving (*10*).

The findings in this report are subject to at least five limitations. First, because NSDUH data are self-reported, they are subject to recall and social desirability biases. Second, variations in laws and regulations among states and counties regarding marijuana could have resulted in negative responses to the NSDUH substance use survey questions for fear of legal consequences, leading to an underestimation of the prevalence of the use and driving under the influence in some jurisdictions. Third, the NSDUH questions are not limited to driving under the influence of marijuana only or each illegal substance only; therefore, persons might be driving under the influence of more than one substance at a given time. Fourth, self-reported data are subject to the respondents’ interpretations of being under the influence of a drug. Finally, NSDUH does not assess whether all respondents drive; therefore, reported percentages of impaired drivers might be underestimated.

Impaired driving is a serious public health concern that needs to be addressed to safeguard the health and safety of all who use the road, including drivers, passengers, pedestrians, bicyclists, and motorcyclists. Collaboration among public health, transportation safety, law enforcement, and federal and state officials is needed for the development, evaluation, and further implementation of strategies to prevent alcohol-, drug-, and polysubstance-impaired driving (*2*). In addition, standardized testing for alcohol and drugs among impaired drivers and drivers involved in fatal crashes could advance understanding of drug- and polysubstance-impaired driving and assist states and communities with targeted prevention efforts.

**Summary**

**What is already known about this topic?**

The use and co-use of alcohol and drugs has been associated with impairment of psychomotor and cognitive functions while driving.

**What is added by this report?**

During 2018, approximately 12 million (4.7%) U.S. residents aged ≥16 years reported driving under the influence of marijuana, and 2.3 million (0.9%) reported driving under the influence of illicit drugs other than marijuana during the past 12 months.

**What are the implications for public health practice?**

Development, evaluation, and further implementation of strategies to prevent alcohol-, drug-, and polysubstance-impaired driving coupled with standardized testing of impaired drivers and drivers involved in fatal crashes could advance understanding of drug- and polysubstance-impaired driving and assist states and communities with prevention efforts.

## Footnotes

* https://crashstats.nhtsa.dot.gov/Api/Public/ViewPublication/812826.

† https://www.cdc.gov/motorvehiclesafety/impaired_driving/strategies.html.

§ https://www.samhsa.gov/data/data-we-collect/nsduh-national-survey-drug-use-and-health. Starting in 2016, NSDUH replaced questions regarding driving under the influence of illicit drugs overall with questions about driving under the influence of individual substances, including cocaine, hallucinogens, heroin, inhalants, marijuana, and methamphetamines.

¶ https://www.cdc.gov/motorvehiclesafety/teen_drivers/teendrivers_factsheet.html.
